# An evaluation of vilobelimab (anti-C5a) as a cost-effective option to treat severely ill mechanically ventilated patients with COVID-19

**DOI:** 10.1093/ajhp/zxae318

**Published:** 2024-10-30

**Authors:** Daniel C Malone, Joseph Biskupiak, Diana Brixner, Gary Oderda, Roger Seheult

**Affiliations:** College of Pharmacy, University of Utah, Salt Lake City, UT, USA; College of Pharmacy, University of Utah, Salt Lake City, UT, USA; College of Pharmacy, University of Utah, Salt Lake City, UT, USA; College of Pharmacy, University of Utah, Salt Lake City, UT, USA; University of California Riverside School of Medicine, Riverside, CA, and Loma Linda University School of Medicine, Loma Linda, CA, USA

**Keywords:** cost-effectiveness analysis, COVID-19, QALY, quality adjusted life years, treatment effectiveness, vilobelimab

## Abstract

**Purpose:**

COVID-19 patients in intensive care units (ICUs) requiring invasive mechanical ventilation (IMV) have few available treatment options. PANAMO, a multicenter, double-blind, randomized, placebo-controlled phase 3 study of vilobelimab, which blocks the inflammatory process caused by complement component 5a, demonstrated a significant mortality benefit at 28 and 60 days in these patients. A cost-effectiveness analysis was conducted to assess the incremental cost per quality-adjusted life-year (QALY).

**Methods:**

A Markov model was used to estimate QALYs and the incremental cost-effectiveness ratio (ICER) of vilobelimab plus standard of care (SOC) versus SOC alone. The model simulated progression from severe COVID-19 to survival or death over a lifetime horizon. Outcomes data (COVID-19 all-cause mortality and renal replacement therapy) were incorporated from the PANAMO trial. COVID-19 mortality estimates were based on Centers for Disease Control and Prevention age-specific survival data. Utility values and hospital costs came from the literature. Vilobelimab cost was obtained from RED BOOK Online.

**Results:**

For COVID-19 ICU patients, total costs of care were $103,414 (SOC) and $132,247 (SOC plus vilobelimab), respectively, resulting in an incremental cost of $28,833. SOC provided 6.70 QALYs versus 7.99 QALYs for vilobelimab, an additional 1.29 QALYs. The ICER for vilobelimab plus SOC versus SOC alone was $22,287/QALY. Probabilistic sensitivity analysis demonstrated the robustness of the cost-effectiveness result as vilobelimab plus SOC was favored at a willingness-to-pay threshold of $50,000 in over 81% of iterations.

**Conclusion:**

Vilobelimab provides a cost-effective option to treat ICU patients with severe COVID-19 receiving IMV compared to SOC, at well below the commonly accepted $50,000 US willingness-to-pay threshold.

**Key Points**

- For critically ill COVID-19 patients receiving invasive mechanical ventilation, vilobelimab provides significant mortality benefit at 28 and 60 days versus standard of care.
- Despite clinical evidence showing vilobelimab has a significant benefit in mortality reduction, hospitals and payers may have reservations treating patients with vilobelimab due to its cost.
- A cost-effectiveness analysis shows adding vilobelimab to standard of care for critically ill COVID-19 patients is cost-effective at a value well below the commonly accepted $50,000 willingness-to-pay threshold.

Infection with severe acute respiratory syndrome coronavirus 2 (SARS-CoV-2) can lead to severe coronavirus disease 2019 (COVID-19) respiratory illness culminating in viral pneumonia, sepsis, and acute respiratory distress syndrome (ARDS).^1,2^ A small number of hospitalized patients with COVID-19 require intensive care. The standard of care (SOC) for these patients includes supplemental oxygen, glucocorticosteroids, antiplatelet agents, and sometimes immunomodulators such as tocilizumab and baricitinib.^3^ However, despite optimal care, case fatality rates are high because of multiorgan failure, which has been explained by secondary damage due to the septic inflammatory host response.^4^

Severe COVID-19 is characterized by inflammation and coagulation in the presence of complement system activation, resulting from the activation of the complement factor 5a (C5a)/C5a receptor 1 (C5aR1) signaling axis.^5,6^ The potent anaphylatoxin C5a and C5aR1 play a critical role in activating and recruiting neutrophils and monocytes to the infection site, causing tissue damage by oxidative radical formation, enzyme release, and induction of NETosis. Tissue factor release is induced from endothelial cells and neutrophils, thereby activating the coagulation system. Thus, C5a has been suggested to play a key role in the development of ARDS in various viral ARDS disease models.^7^

Vilobelimab (Gohibic) is a chimeric monoclonal immunoglobulin G4 antibody that specifically binds with high affinity to the soluble form of human C5a. The potential benefit of vilobelimab was investigated in the adaptively designed phase 2 and phase 3 PANAMO study, which confirmed the efficacy and safety of vilobelimab in selectively blocking C5a in patients with severe pneumonia induced by COVID-19 requiring IMV when treatment was administered within 48 hours.^8^ The results of the PANAMO phase 3 study demonstrated a significant survival benefit in the vilobelimab plus SoC treatment arm (n = 177) compared to the placebo plus SOC arm (n = 191) for the primary endpoint of 28-day all-cause mortality (a 23.9% relative risk reduction) as well as for 60-day all-cause mortality as a key secondary endpoint (a 22.7% relative risk reduction).^8^ The Kaplan-Meier estimate of the 28-day mortality rate in the vilobelimab group was 31.7%, and the estimated rate in the placebo group was 41.6%, resulting in a hazard ratio (HR) of 0.67 (95% CI, 0.48-0.96; *P* < 0.05), as calculated using non–site-stratified, age-adjusted Cox regression analysis. Results were similar at day 60.^8^ All-cause mortality at 60 days was observed in 62 (35%) of 177 patients in the vilobelimab group and in 87 (46%) of 191 in the placebo group (HR, 0.67; 95% CI, 0.48-0.93; *P* < 0.05).

The purpose of this study is to describe the methods and results of a cost-effectiveness model of vilobelimab added to SOC based on the phase 3 PANAMO clinical study data.

## Methods

**Figure 1.**
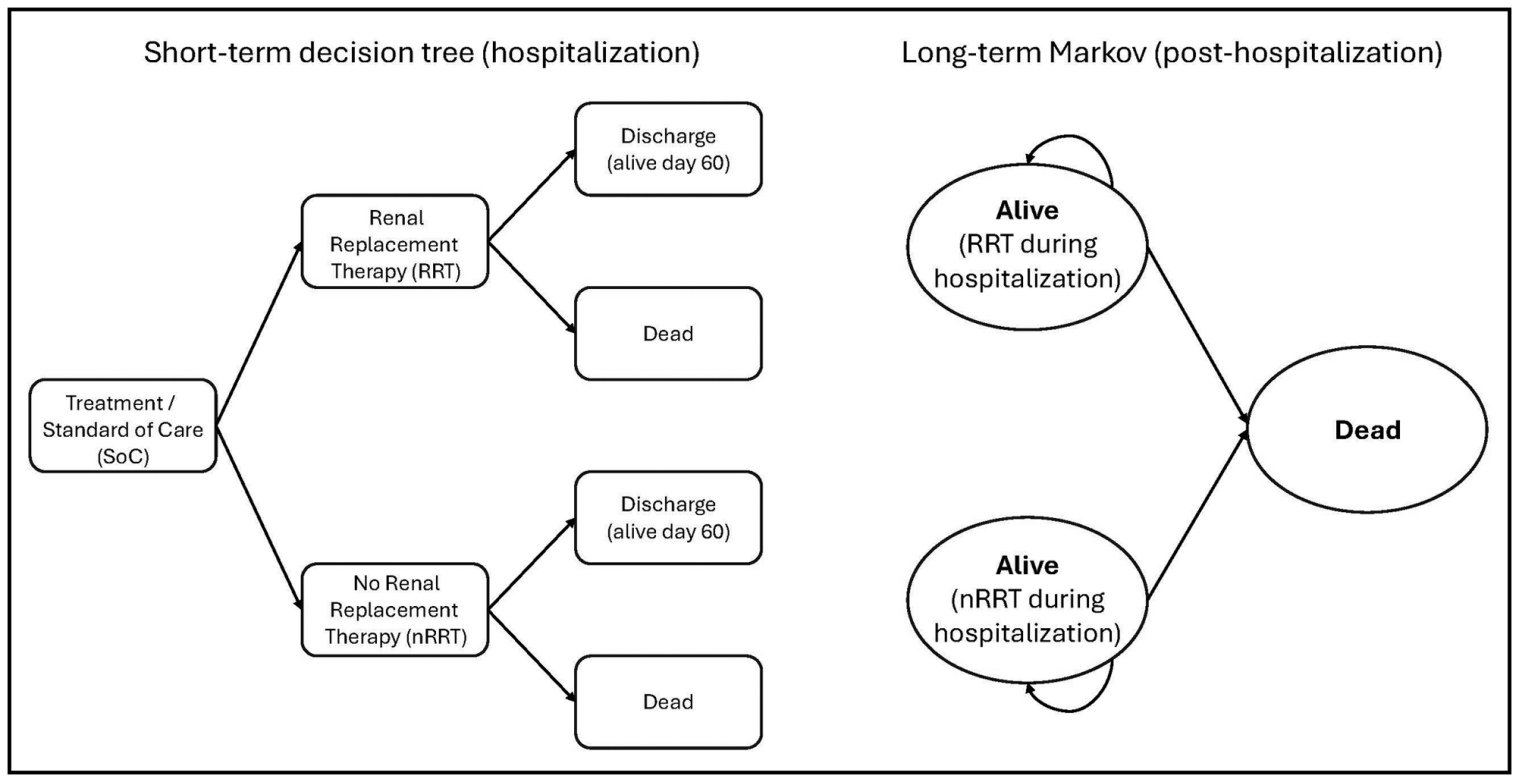
Cost-effectiveness model structure used for the analysis, showing short-term decision tree (hospitalization) and long-term Markov model (posthospitalization).

A cost-effectiveness model was developed using TreeAge Pro 2023 (TreeAge Software, LLC, Williamstown, MA) to estimate the impact of the addition of vilobelimab to SOC relative to SOC alone for hospitalized patients with severe COVID-19 requiring IMV. A lifetime model was conducted from a modified societal perspective. The model included a short-term acute care decision tree followed by a postdischarge 2-state Markov cohort model with a cycle length of 1 month and half-cycle correction (Figure 1). The model estimated progression from severe COVID-19 to survival or death and the receipt of renal replacement therapy. Survivors then transitioned to the Markov model, in which the transition probability from alive to death was based on Centers for Disease Control and Prevention (CDC) life table values.^9^ This model structure was chosen to best utilize the clinical trial data (inpatient acute stay) and the lifetime aspects of the model.

The short-term acute care decision tree was derived from the clinical outcomes data from the phase 3 PANAMO study, which demonstrated a survival benefit with the addition of vilobelimab to SOC versus SOC alone at days 28 and 60.^8^ Further, the use of renal replacement therapy was found to differ significantly between the two treatments in the study. All other resource utilization (including inpatient length of stay [LOS], ICU LOS, and ventilation days) and adverse events (AEs) (including treatment-emergent AEs and serious treatment-emergent AEs) were similar between the treatments and not included in the model. Patients who survived the acute episode (ie, patients alive on day 60) then transitioned to the Markov model. Costs and health effects were discounted at 3% per year. Key effectiveness measures included total life-years, quality-adjusted life-years (QALYs), and equal value of life-years gained (evLYG). A willingness-to-pay (WTP) threshold of $50,000/QALY was used.^10^ The evLYG values all gains in life-years at the full value of a healthy life-year, such that regardless of age, disability, or illness, all life-year gains are valued equally. The incremental cost-effectiveness ratio (ICER) utilizes a value of 0.851 for the value of a healthy life-year based on the age- and gender-adjusted utility of the healthy US population.^11^

Clinical outcomes data for patients treated with vilobelimab and SOC were derived from the phase 3 PANAMO study and are shown in Table 1.^8^ The 60-day all-cause mortality data was used to capture all the survival data available from the trial. At day 60, 36.5% of the patients in the vilobelimab plus SOC cohort were deceased, compared to 47.2% of patients in the SOC group. Vilobelimab plus SOC was protective against renal replacement therapy at day 28 (age-adjusted HR, 0.54 [95% CI, 0.30-0.98]; *P* = 0.04). Additional parameters for the model were from a study by Sheinson et al,^16^ who described a cost-effectiveness framework for hospitalized COVID-19 patients, and from CDC Life Tables^9^ (Table 2). Cost inputs are also shown in Table 2. Renal replacement therapy costs ($2,000/day) were as reported by Tseng et al^12^ and expressed in 2023 US dollars (inflated using the medical care services component of the Consumer Price Index). The cost of vilobelimab was based on the wholesale acquisition price as listed in Micromedex RED BOOK Online.^13^ The cost of infusion was not considered in this analysis because all patients were hospitalized and receiving other supportive care. The cost for SOC was set at $100,461 for this patient population and was derived from a study from the Kaiser Family Foundation,^14^ similar to results by Bazell et al.^17^ Because this cost was applied equally to both treatment groups, it was not inflated to 2023 US dollars. Annual healthcare costs for survivors were not included in the model. In the base case, patients entered the model at age 56.3 years based on trial data. To assess the robustness of the model results, both deterministic and probabilistic sensitivity analyses were conducted. Estimates based on the PANAMO study used 95% CI values for the lower and upper bounds of the sensitivity analysis.

**Table 1.** Clinical Outcomes Data and Cost Estimates Used in the Analysis

	Base case	Range	Distribution	Source
**Clinical outcomes**				
Vilobelimab 60-day survival	0.65	0.58-0.73	Beta	Vlaar et al^8^
Standard of care 60-day survival	0.48	0.54-0.62	Beta	Vlaar et al^8^
Renal replacement therapy for vilobelimab	0.096	0.086-0.106	Beta	Vlaar et al^8^
Renal replacement therapy for standard of care	0.157	0.141-0.173	Beta	Vlaar et al^8^
Duration of renal replacement therapy for vilobelimab	6.8	7.8-8.8	Gamma	Vlaar et al^8^
Duration of renal replacement therapy for standard of care	9.4	8.1-10.7	Gamma	Vlaar et al^8^
**Cost estimates**				
Renal replacement therapy	$2,000/day	$1,800-$2,200	NA	Tseng et al^12^
Vilobelimab per administration	$6,350	$5,715-6985	NA	RED BOOK^13^
Intensive care treatment of COVID-19	$100,461	$40,218-$100,461	NA	Kaiser Family Foundation report^14^
Premature mortality	$146,494	NA	NA	Putri et al^15^

Abbreviations: COVID-19, coronavirus disease 2019; NA, not applicable.

**Table 2.** US Age-Specific Utility Values and Disutility Values for Mechanical Ventilation and Postdischarge Disutility for Patients Requiring Mechanical Ventilation Applied for 4 Years^12^

	Base case	SD	Distribution
US age-specific utility			
40-49	0.870	0.002	Beta
50-59	0.840	0.003	Beta
60-69	0.820	0.003	Beta
70-79	0.790	0.004	Beta
>80	0.740	0.006	Beta
Mechanical ventilation during hospitalization disutility	0.560	0.300	Beta
Postdischarge disutility for patients requiring mechanical ventilation			
Year 1	0.130	0.013	Beta
Year 2	0.067	0.007	Beta
Year 3	0.062	0.006	Beta
Year 4	0.026	0.001	Beta

Because a societal perspective was used in the analysis, a secondary analysis that included the cost of premature mortality was conducted. There are no estimates of the cost of premature mortality due to COVID-19 that are specific to the United States. Therefore, we used an estimate of premature mortality from an analysis focused on the influenza virus and updated the estimate to 2023 dollars ($146,494).^15^

As this was a modeling exercise, no institutional review board approval was necessary (and thus no number was assigned) because this did not fall under the board’s guidelines as human subjects research. Only data from clinical trial results that have been published previously were used. The research was conducted according to the principles of the Declaration of Helsinki, seventh revision.^18^

## Results

The base case analysis describes the cost-effectiveness of treating hospitalized patients with severe COVID-19 requiring IMV (Table 3). Use of vilobelimab plus SOC resulted in 7.99 QALYs, as compared to 6.70 QALYs with SOC alone. Associated costs were $132,247 with vilobelimab plus SOC and $103,414 with SOC alone. The ICER for vilobelimab plus SOC compared with SOC alone was $22,287/QALY. In addition, the use of vilobelimab plus SOC gained 15.18 life-years and 9.03 evLYG, compared to 12.72 life-years and 7.57 evLYG with SOC alone. In a secondary analysis that included the cost of premature mortality due to COVID-19, the resulting cost was $183,561 for vilobelimab plus SOC and $170,142 for SOC alone. The resulting ICER was $10,373 per QALY for vilobelimab plus SOC compared with SOC alone.

**Table 3.** Results of Base Case Sensitivity Analysis

Strategy	Cost	Incremental cost	QALYs	Incremental effect (QALYs)	ICER
Standard of care	$103,414	[Reference]	6.70	[Reference]	[Reference]
Vilobelimab	$132,247	$28,833	7.99	1.29	$22,287

Abbreviations: ICER, incremental cost-effectiveness ratio; QALY, quality-adjusted life-year.

**Figure 2.**
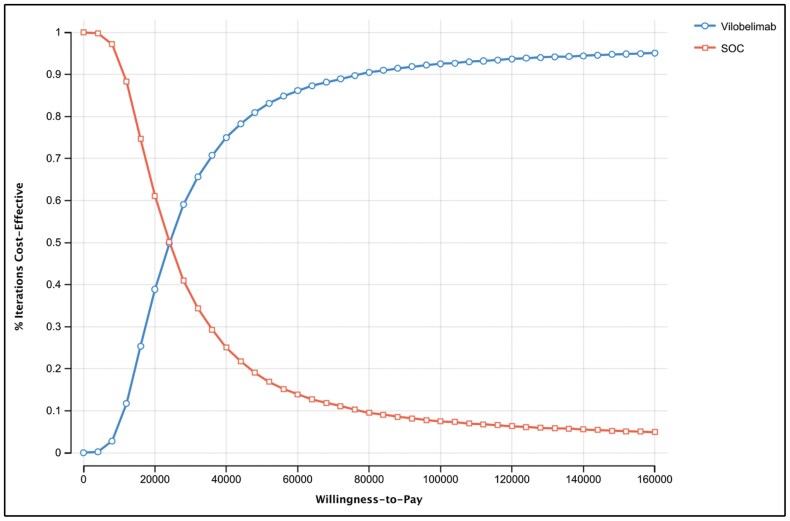
Cost-effectiveness acceptability curve of vilobelimab versus standard of care (SOC) at a willingness-to-pay threshold of $50,000.

**Figure 3.**
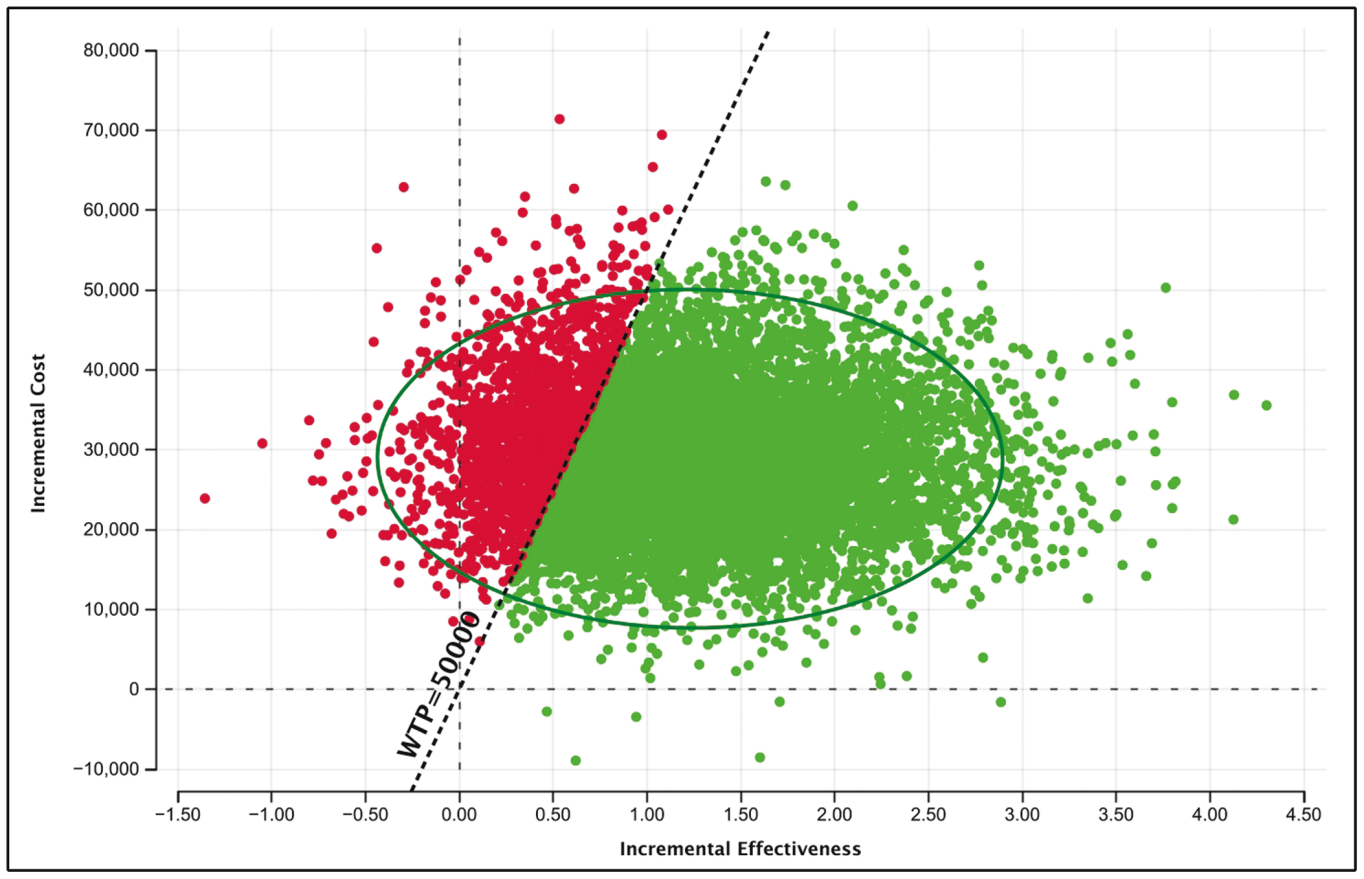
Incremental cost-effectiveness ratio (ICER) scatterplot of vilobelimab versus standard of care (SOC), with a willingness-to-pay threshold (WTP) of $50,000.

Probabilistic sensitivity analysis (PSA) demonstrated that the cost-effectiveness results were highly robust to uncertainty. The PSA was run with 10,000 draws to produce stable results. Greater than 81% of draws had a cost per QALY gained below the $50,000 WTP threshold (Figures 2 and 3). Further demonstrating the robustness of the results, the cost-effectiveness acceptability curve (CEAC) shows that at a WTP threshold of $100,000, greater than 92% of the draws had a cost per QALY gained below the threshold (Figure 3).

**Figure 4.**
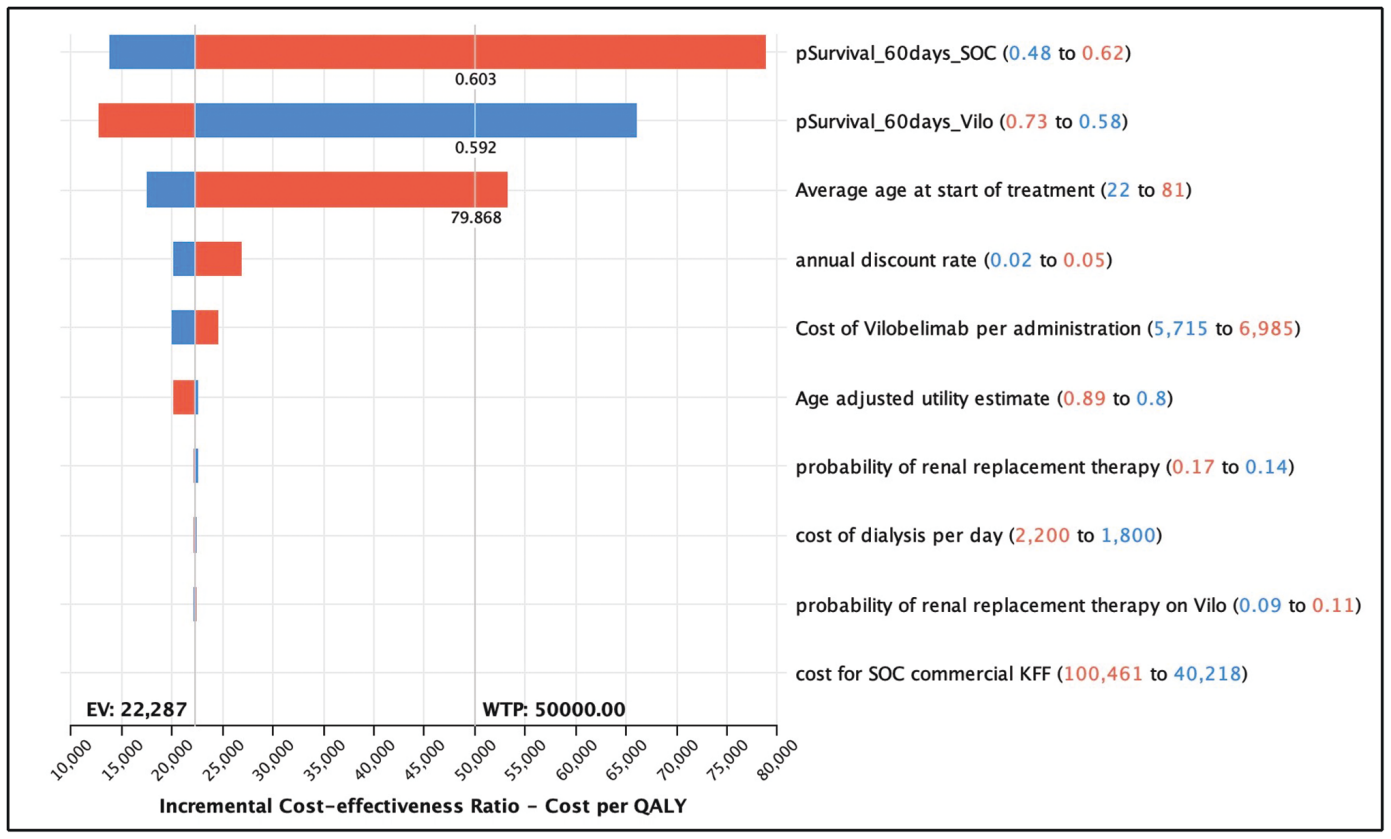
Tornado diagram for vilobelimab versus standard of care (SOC), showing the parameters with the greatest impact on the incremental cost-effectiveness ratio were survival rate and age.

One-way sensitivity analysis (Table 4 and Figure 4) found that the parameters with the greatest impact on the ICER were survival rate (baseline value, 62.5% [range, 55%-69%] with vilobelimab and 48% [range, 45%-60%] with SOC) and age (baseline value, 56.3 years; range, 22-81 years). The ICER of vilobelimab plus SOC compared with SOC alone increased with decreasing survival probability with use of vilobelimab plus SOC, increasing age, and increasing survival probability with use of SOC alone. In the worst-case scenario (ie, the lower bound of survival probability for vilobelimab plus SOC), the ICER was $66,058/QALY. In the best-case scenario of the upper bound of survival probability for vilobelimab plus SOC, the ICER was $12,641/QALY.

**Table 4.** Results of One-way Sensitivity Analysis (Top 3)

Variable	Variable value			Impact	ICER	
	Low	Base	High		Low	High
Survival probability at day 60 with SOC	0.48	0.54	0.62	Increase	$13,816	$78,905
Survival probability at day 60 with vilobelimab + SOC	0.58	0.65	0.73	Decrease	$66,058	$12,641
Age	22	56.3	81	Increase	$17,470	$53,195

Abbreviation: ICER, incremental cost-effectiveness ratio.

## Discussion

Currently, critically ill COVID-19 patients hospitalized and requiring IMV have few treatment options beyond supportive care. However, the US Food and Drug Administration has issued an emergency use authorization (EUA) for the emergency use of Gohibic (vilobelimab) for the treatment of COVID-19 in hospitalized adults when initiated within 48 hours of receiving IMV or extracorporeal membrane oxygenation (ECMO).^19-21^

Despite the clinical evidence that demonstrates vilobelimab has a significant benefit in mortality reduction, there is concern that hospitals and payers may have reservations about treating patients with vilobelimab due to its cost and the use of diagnosis-related groups (DRGs) to determine reimbursement payments. The additional 1.29 QALYs gained for COVID-19 patients requiring IMV and receiving vilobelimab with SOC versus SOC alone is a large gain and drives the acceptable ICER threshold of $22,287 per patient treated. The results of the reported cost-effectiveness model for vilobelimab added to SOC versus SOC alone demonstrated that vilobelimab is cost-effective in comparison to widely accepted US cost-effectiveness thresholds (currently at $50,000 for cost-effective treatments and $100,000 for moderately cost-effective treatments; values above $150,000 per QALY are considered not cost-effective). In addition, for rare diseases, thresholds as high as $175,000 have been accepted.^22^ Of note, the authorized use of vilobelimab is restricted to a critically ill patient population of adult COVID-19 patients requiring IMV or ECMO and, as estimated based on reported CDC numbers, reflects patient numbers far below the threshold set in the US for orphan diseases.

There has been some controversy concerning the use of vilobelimab because of the statistical analysis. The protocol initially agreed upon by the sponsor and FDA specified a non–site-stratified approach within Cox regression for the phase 3 PANAMO study. During the latter part of the study, FDA recommended investigational site stratification using Cox regression to account for potential cross-site heterogeneity. This approach was adopted by the sponsor and thus became the prespecified method for the analysis. Using site stratification within Cox regression to analyze the PANAMO data led to approximately 17% (n = 61) of all enrolled patients being excluded from contributing to the analyzed output due to lack of events (ie, deaths) at several sites (n = 55) or only death at 6 placebo single patient enrollment sites (n = 6). This exclusion led to a loss of power reflected in the resulting *P* value (0.094). Upon further analysis using stratification by country, region, and pooling small enrollment sites, FDA within its review of vilobelimab recognized this limitation and adopted the originally proposed method (Cox regression without site-stratification adjustment) as the “more reliable” method (*P* = 0.026) and stated that “there were no data integrity issues,” ultimately concluding that a significant mortality benefit had been demonstrated.^20^ This was the basis for an EUA for Gohibic in the US for the treatment of COVID-19 in hospitalized adults when initiated within 48 hours of receiving IMV or ECMO.^23^ SARS-CoV-2 infections continue in the US, but severe COVID-19 requiring IMV has decreased dramatically in the last year due to widespread vaccination and infection. This has resulted in fewer patients being admitted to an ICU. Though vilobelimab received a neutral recommendation from the National Institutes of Health (NIH) COVID-19 panel, uptake of the product is occurring in the rare circumstances where COVID-19 patients are intubated. The NIH COVID-19 panel has disbanded, and the website for the guidelines was scheduled for termination on August 16, 2024.^24^

Other treatment options for hospitalized COVID-19 patients include barcitinib,^25^ tocilizumab,^26^ and remdesivir,^27^ but those treatments are not specific to IMV patients. The NIH COVID-19 panel’s guidelines states that studies used for authorization of tocilizumab and baricitinib were “not specifically powered” and had “a lower certainty of evidence,” respectively, in patients requiring IMV or ECMO^24^ compared to the phase 3 PANAMO study of vilobelimab, which was a priori powered to assess a difference in 28-day all-cause mortality in IMV or ECMO patients.^8^ In a model that simulated an inpatient stay, barcitinib provided a modest increase in survival (5.1%) relative to SOC, at $25,774/QALY. This model was based on a study that excluded COVID-19 patients on IMV but included hospitalized patients who primarily required low- or high-flow oxygen.^28^ Tocilizumab provided a 4% survival benefit in the open-label platform RECOVERY trial, and published cost-effectiveness model results indicated an ICER of $16,520 per QALY compared to SOC.^29^ In a cost-effectiveness analysis of remdesivir for hospitalized COVID-19 patients with no survival benefit, the incremental cost-effectiveness was $298,200/QALY for patients with moderate to severe COVID-19 and $1,847,000/QALY for patients with mild COVID-19.^30,31^

### Limitations

 This model uses a modified societal perspective, although data on posthospital productivity was not included because this information was not available from the PANAMO study. A lifetime perspective was utilized, although mortality data was only available for 28 and 60 days. Patients who were discharged from the hospital during the study were assumed to have a similar life expectancy based on their discharge age, although disutility values were applied for the first 5 years because all patients required mechanical ventilation. Hospital costs were assumed to be the same in both groups except for the cost of vilobelimab and renal replacement therapy. The model also assumes that long-term life expectancy is not affected by receiving IMV due to COVID-19.

Additional limitations are based on the data used to populate the model, which was supported by a clinical study that was conducted outside of the US. The model inputs were also subject to the restrictions of a clinical study. These limitations can be addressed as real-world data on the utilization, outcomes, and costs of vilobelimab become available post FDA approval.

## Conclusion

Relative to SOC, vilobelimab provides a cost-effective option to treat patients with severe COVID-19 who are receiving IMV, at a value well below the commonly accepted US WTP threshold of $50,000.

## Data Availability

All data for the model was derived from published literature sources and refrenced accordingly
